# Laparoscopic distal gastrectomy for synchronous adenocarcinoma, diffuse large B cell lymphoma and gastrointestinal stromal tumor in the stomach: a case report

**DOI:** 10.1186/s40792-022-01446-1

**Published:** 2022-05-08

**Authors:** Toshiyasu Ojima, Hirotaka Tabata, Hiroki Yamaue

**Affiliations:** Second Department of Surgery, School of Medicine, Wakayama Medical University, 811-1, Kimiidera, Wakayama, 641-8510 Japan

**Keywords:** Adenocarcinoma, Diffuse large B cell lymphoma, Gastrointestinal stromal tumor

## Abstract

**Background:**

Synchronous lymphoma and adenocarcinoma are occasionally detected in the stomach. Gastrointestinal stromal tumor (GIST) and adenocarcinoma are sometimes seen simultaneously in the stomach. However, we rarely observe synchronous adenocarcinoma, lymphoma, and GIST in the stomach, and there are few reports on cases with these three lesions.

**Case presentation:**

This is a case report of a 71-year-old man who had a laparoscopic distal gastrectomy and lymphadenectomy for three gastric tumors. Preoperative diagnoses were early adenocarcinoma and mucosa-associated lymphoid tissue (MALT) lymphoma in the stomach, but final diagnosis was synchronous adenocarcinoma, diffuse large B cell lymphoma (DLBCL), and GIST. *Helicobacter pylori* (*H. pylori*) is highly involved in the development of DLBCL and MALT lymphoma in the stomach. Gastric adenocarcinoma is partially involved in chronic gastritis with atrophy and intestinal metaplasia caused by *H. pylori* infection. Indeed, a rapid urease test was found positive in this case. Therefore, we prescribed medicine to eliminate *H. pylori* after gastrectomy.

**Conclusion:**

This is the first case report where a patient underwent minimally invasive laparoscopic gastrectomy for synchronous adenocarcinoma, DLBCL and GIST in the stomach, although one patient who underwent open gastrectomy for synchronous adenocarcinoma, MALT lymphoma and GIST was previously reported.

## Background

Gastric lymphoma constitutes 3–6% of primary gastric tumor [1, 2]. Gastric lymphoma, especially mucosa-associated lymphoid tissue (MALT) lymphoma, is associated with *Helicobacter pylori* (*H. pylori*). In some cases, synchronous lymphoma and adenocarcinoma are also detected in the stomach. Gastrointestinal stromal tumor (GIST) is most commonly seen in the stomach (60–70%) [2]. Therefore, GIST and adenocarcinoma are sometimes seen simultaneously in the stomach. However, we rarely observe synchronous adenocarcinoma, lymphoma, and GIST in the stomach, and there are few reports on cases with these three lesions [1]. Here, we present a case with adenocarcinoma, diffuse large B cell lymphoma (DLBCL), and GIST simultaneously observed in the stomach. This is the first report of a patient who underwent laparoscopic gastrectomy for synchronous adenocarcinoma, DLBCL and GIST in the stomach.

## Case presentation

**Fig. 1:**
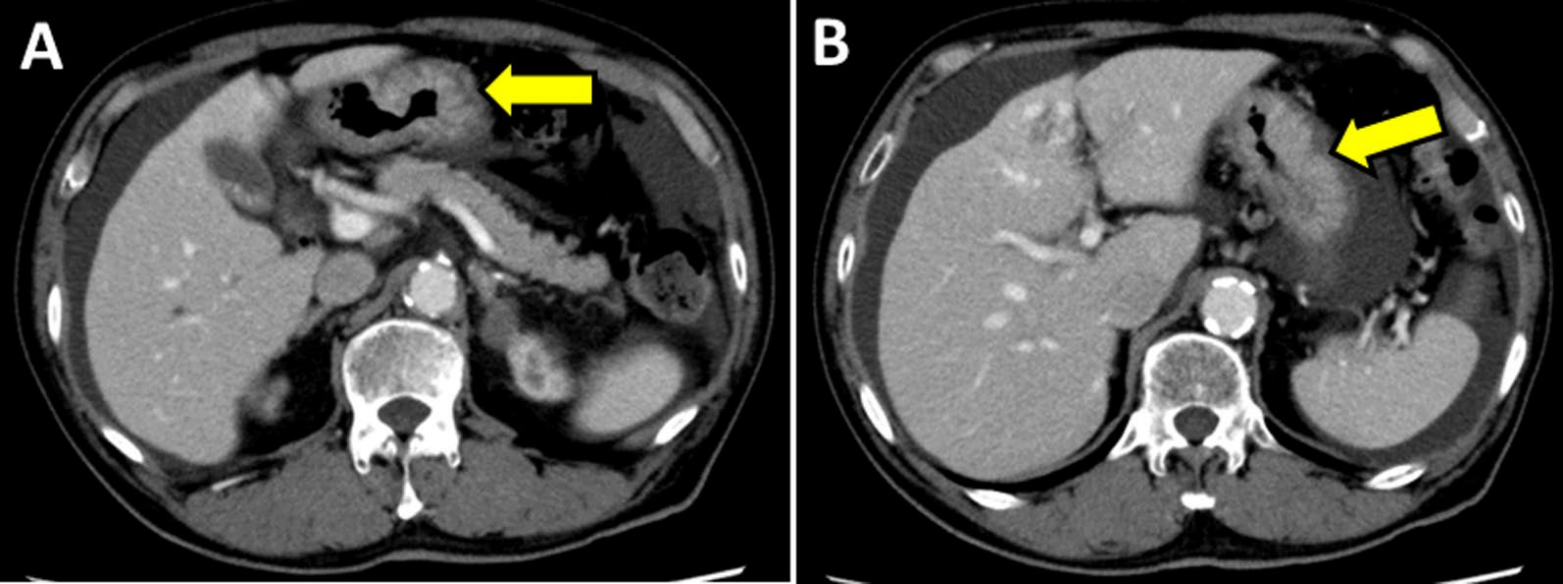
Computed tomography scan. Yellow arrows show the gastric wall thickness in the lower gastric body. **A** Gastric cancer, **B** lymphoma

**Fig. 2:**
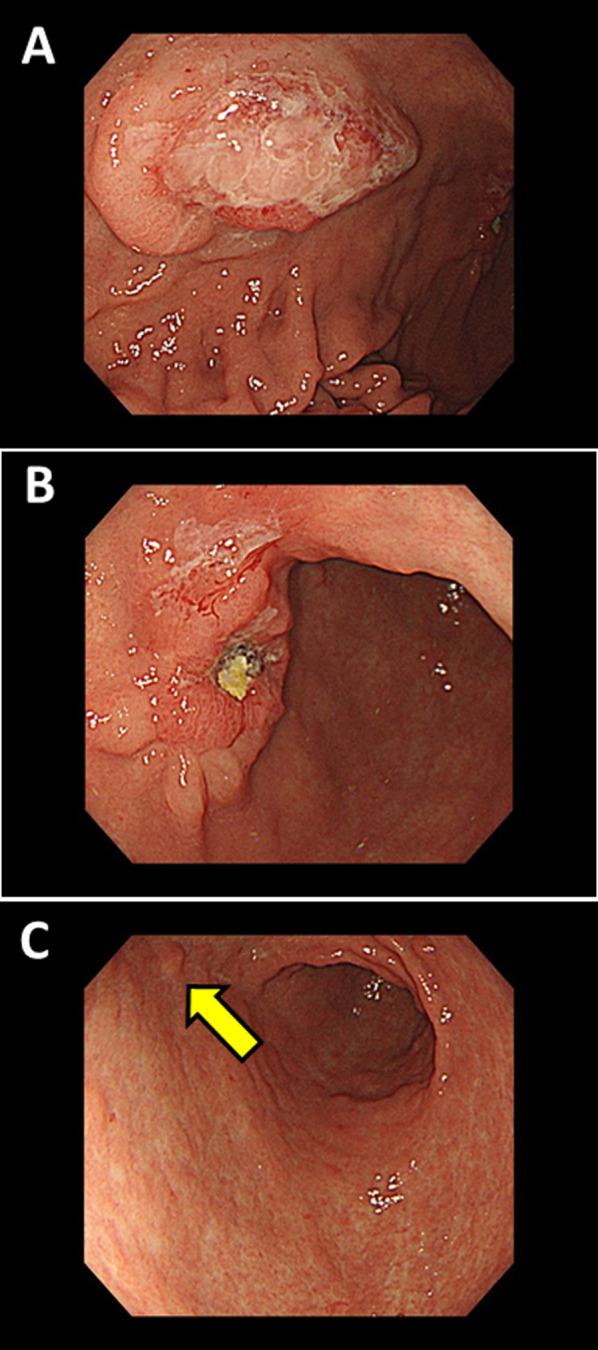
Gastrointestinal endoscopy findings. **A** Lymphoma, **B** gastric cancer, **C** submucosal tumor

A 71-year-old man was admitted to our hospital for hematemesis and anemia. He had conjunctival anemia. Other physical examination was normal. Abnormal laboratory values included hemoglobin level at 9.5 g/dL and hematocrit level at 30.9%. Gastric wall thickness was detected in the lower gastric body in abdominal computed tomography scan (Fig. 1). Gastrointestinal endoscopy demonstrated type 2 lesion with a maximal size of 32 mm in the anterior side of the lower gastric body (Fig. 2A) and type 0–IIa + IIc lesion with a maximal size of 24 mm in the anterior side of the gastric angle (Fig. 2B). The latter lesion was bleeding and electric cauterization was performed to stop the bleeding. In addition, a small submucosal tumor was found in the antrum region of the stomach (Fig. 2C). On hospital day 8, second gastrointestinal endoscopy was performed and biopsies were taken from both lesions. Rapid urease test was positive. Histological examination of the gastric body revealed MALT lymphoma and the lesion in the gastric angle was diagnosed with poorly differentiated adenocarcinoma. The preoperative diagnosis of gastric adenocarcinoma was cT1b cN0 cStage IA (8th TNM classification).

**Fig. 3:**
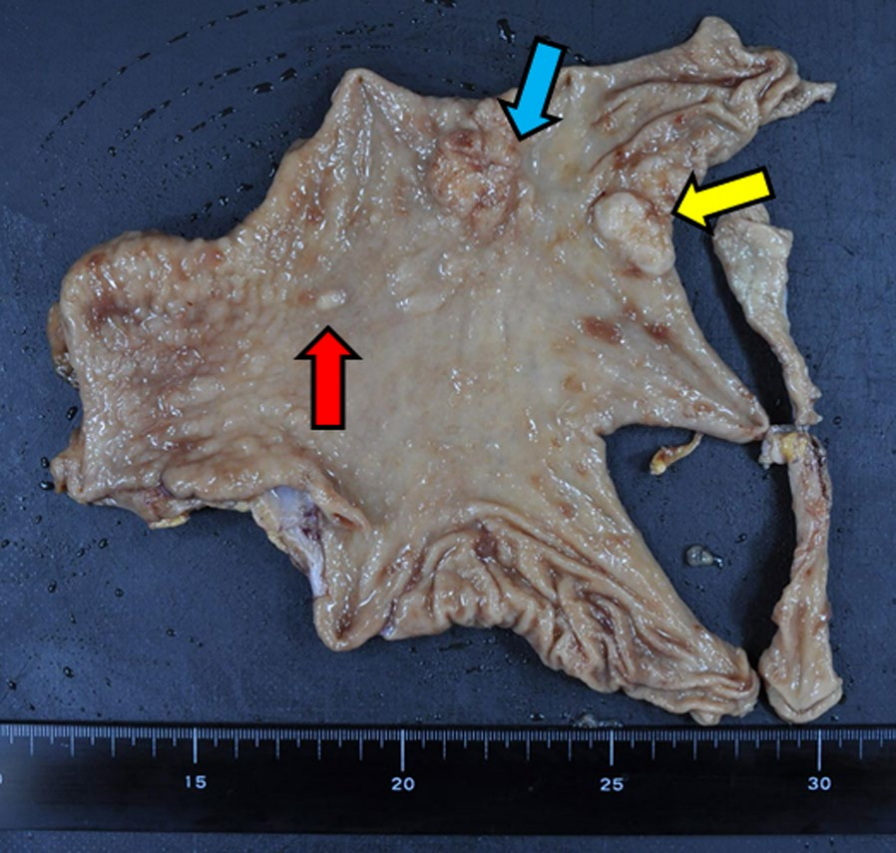
Resected specimen. Blue arrow shows the gastric cancer. Yellow arrow shows the lymphoma. Red arrow shows the submucosal tumor

We performed laparoscopic distal gastrectomy with D1 + lymph node dissections and R-Y reconstruction (Fig. 3). The operation was successfully performed with uneventful outcome.

**Fig. 4:**
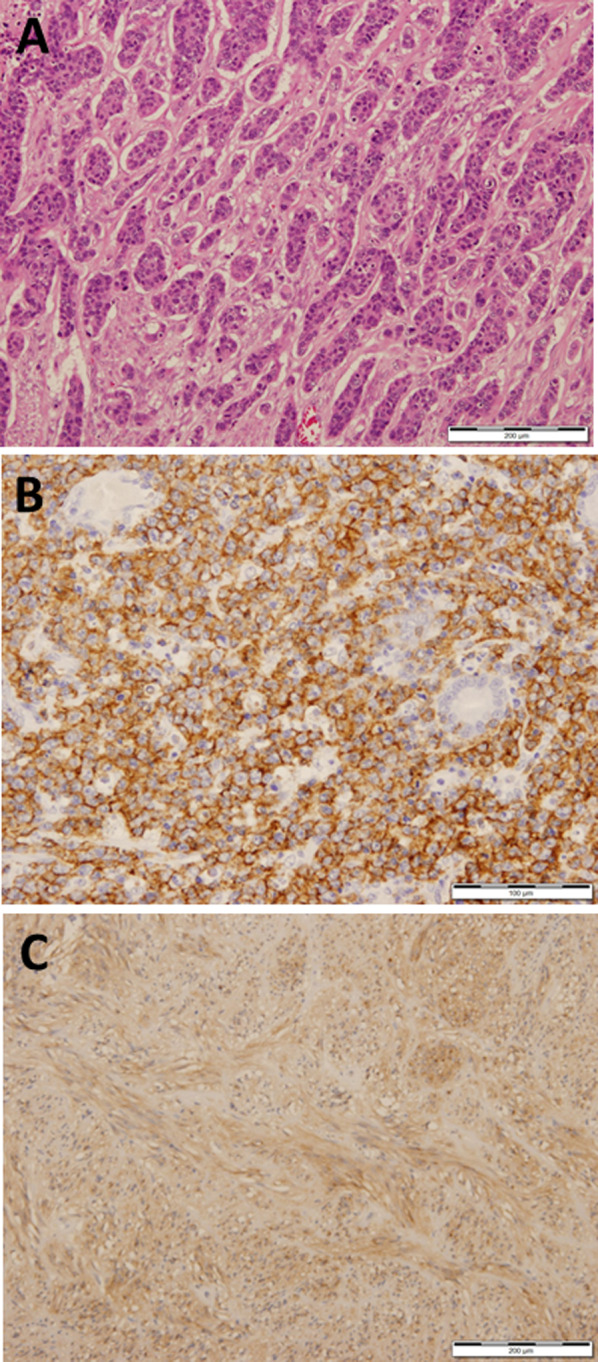
Histological findings. **A** Poorly differentiated adenocarcinoma infiltrating into deep submucosa (hematoxylin–eosin: × 100). **B** Diffuse large B cell lymphoma tissue containing large atypical lymphocytes immunostaining for CD20 (× 200). **C** Gastrointestinal stromal tumor tissue containing with spindle, sharp and atypical cell immunostaining for c-kit (× 100)

Histological findings of the sample taken from the tumor in the gastric angle confirmed the diagnosis of poorly differentiated adenocarcinoma (Fig. 4A) which infiltrated into deep submucosa. MALT lymphoma was the preoperative diagnosis of the tumor in the lower gastric body; however, postoperative histological findings confirmed the diagnosis of DLBCL. Large diffused atypical lymphocytes were observed in whole layer of the lower gastric body, and the tumor cells were stained with bcl-6, CD10 and CD20 (Fig. 4B). In the tumor tissue, lymphoepithelial lesion (LEL) of characteristic of MALT lymphoma was observed, but strong atypical lymphocytes and high level of MIB-1 (70–80%) were observed. Taken together, the final diagnosis of the tumor in the lower gastric body was DLBCL. In addition, a 1.0-cm nodule was found on the serosal surface of the anterior wall of the antrum of the stomach in the process of histocytological preparation. Sections of the serosal nodule showed a GIST composed of uniform spindle cells. Immunostaining for CD34 and c-kit were positive (Fig. 4C) while S-100 and desmin were negative. GIST was classified as very low risk in the Fletcher risk table. No perigastric lymph nodes contained malignant component was found. The patient had an uneventful postoperative recovery and was discharged with a good clinical prognosis. We prescribed medicine to eradicate *H. pylori* after gastrectomy.

## Discussion

In this case, the preoperative diagnoses were synchronous gastric adenocarcinoma and gastric MALT lymphoma. Generally, MALT lymphoma is the most common (50–60%) while DLBCL is the second most common (40–50%) in the stomach [3]. *H. pylori* infection is known to be highly involved in the development of MALT lymphoma [4] and the first-line therapy for MALT lymphoma is the eradication of *H. pylori*. In addition, it has been reported that DLBCL may comprise a component of MALT lymphoma [5]. In this case, LEL was observed and it was highly relevant for the diagnosis of gastric MALT lymphoma. For this reason, we considered the DLBCL in this case had a component of MALT lymphoma and the development of the DLBCL may potentially involve *H. pylori* infection (Table 1). The development of gastric adenocarcinoma partially contributed to chronic gastritis with atrophy and intestinal metaplasia caused by *H. pylori* infection (Table 1).

**Table 1:** Two cases with adenocarcinoma, lymphoma, and GIST in the stomach

Year	Gender	Histology	Stage*	*H. pylori*	Treatment	Chemotherapy	Prognosis	Reference
78	Male	Adenocarcinoma MALT lymphoma GIST	IA I IA	Positive	Open total gastrectomy	None	Unknown	1
71	Male	Adenocarcinoma DLBCL GIST	IA I IA	Positive	Laparoscopic distal gastrectomy	None	72 months**	Our case

*8th TNM classification **Recurrence-free survival

Although the stomach is the most common site of involvement of GIST (60–70%), synchronous adenocarcinoma and GIST of the stomach is relatively rare. The development of GIST involves activating mutations in exon 11 of the KIT gene that encodes a tyrosine kinase receptor [6], and there is no evidence linking GIST to *H. pylori* infection at present.

## Conclusion

Although there is one previous case report with synchronous adenocarcinoma, MALT lymphoma and GIST where the patients underwent open gastrectomy were published (Table 1) [1], this is the first report of a patient who underwent laparoscopic gastrectomy for synchronous adenocarcinoma, DLBCL and GIST in the stomach.

## Data Availability

Data sharing is not applicable to this article, as no datasets were generated or analyzed during the current study.

## Abbreviations

MALT: Mucosa-associated lymphoid tissue
*H. pylori*: *Helicobacter pylori*
GIST: Gastrointestinal stromal tumor
DLBCL: Diffuse large B cell lymphoma
LEL: Lymphoepithelial lesion
